# 3-Bromo-9-(4-fluoro­benz­yl)-9*H*-carbazole

**DOI:** 10.1107/S160053680901976X

**Published:** 2009-06-17

**Authors:** Cheng-Feng Wang

**Affiliations:** a College of Chemistry & Bioengineering, Changsha University of Science & Technology, Changsha 410076, People’s Republic of China

## Abstract

The title compound, C_19_H_13_BrFN, was synthesized by *N*-alkyl­ation of 1-chloro­methyl-4-fluoro­benzene with 3-bromo-9*H*-carbazole. The carbazole ring system is essentially planar (r.m.s. deviation of 0.024 Å for the non-H atoms) and forms a dihedral angle of 88.2 (3)° with the benzene ring.

## Related literature

For a similar structure, see: Huang *et al.* (2007 ▶). For the synthetic procedure, see: Duan *et al.* (2005*a* ▶,*b* ▶).
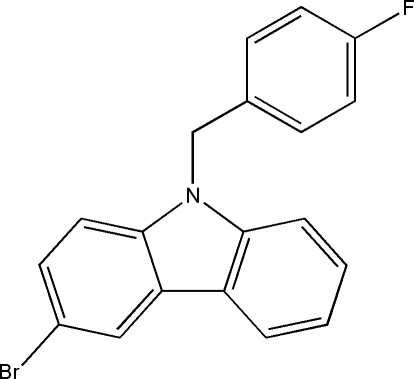


## Experimental

### 

#### Crystal data

C_19_H_13_BrFN
*M* *_r_* = 354.21
Orthorhombic, 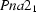

*a* = 17.407 (4) Å
*b* = 15.068 (3) Å
*c* = 5.5865 (11) Å
*V* = 1465.3 (5) Å^3^
*Z* = 4
Mo *K*α radiation
μ = 2.81 mm^−1^
*T* = 113 K
0.18 × 0.12 × 0.08 mm

#### Data collection

Rigaku Saturn diffractometer
Absorption correction: multi-scan (*CrystalClear*; Rigaku/MSC, 2005 ▶) *T*_min_ = 0.632, *T*_max_ = 0.806
9581 measured reflections
2577 independent reflections
2294 reflections with *I* > 2σ(*I*)
*R*_int_ = 0.050

#### Refinement

*R*[*F* ^2^ > 2σ(*F* ^2^)] = 0.032
*wR*(*F* ^2^) = 0.070
*S* = 0.99
2577 reflections
199 parameters
1 restraint
H-atom parameters constrained
Δρ_max_ = 0.43 e Å^−3^
Δρ_min_ = −0.69 e Å^−3^
Absolute structure: Flack (1983 ▶), 1139 Friedel pairs
Flack parameter: 0.004 (12)

### 

Data collection: *CrystalClear* (Rigaku/MSC, 2005 ▶); cell refinement: *CrystalClear*; data reduction: *CrystalClear*; program(s) used to solve structure: *SHELXS97* (Sheldrick, 2008 ▶); program(s) used to refine structure: *SHELXL97* (Sheldrick, 2008 ▶); molecular graphics: *SHELXTL* (Sheldrick, 2008 ▶); software used to prepare material for publication: *SHELXTL*.

## Supplementary Material

Crystal structure: contains datablocks I, global. DOI: 10.1107/S160053680901976X/gk2211sup1.cif

Structure factors: contains datablocks I. DOI: 10.1107/S160053680901976X/gk2211Isup2.hkl

Additional supplementary materials:  crystallographic information; 3D view; checkCIF report

## Figures and Tables

**Fig. 1.**
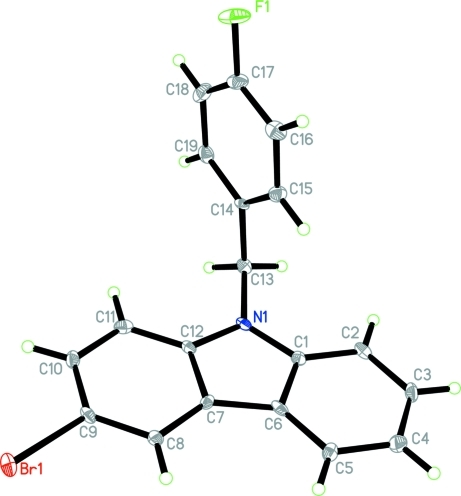
Molecular structure of the title compound with displacement ellipsoids drawn at the 30% probability level. H atoms are presented as spheres of arbitrary radius.

## supplementary crystallographic information

### Comment

N-Alkyl carbazoles possess valuable pharmaceutical properties. In this paper, synthesis and the crystal structure of 3-bromo-9-(4-fluorobenzyl)-9*H*-carbazole is reported

The carbazole ring is essentially planar, with a r.m.s. deviation from the mean plane of 0.024 Å for the non-hydrogen atoms. The dihedral angle formed between the carbazole unit and the benzene ring is 88.2 (3) Å.

### Experimental

The title compound was prepared according to the procedure of Duan *et al.* (2005a,b). A solution of potassium hydroxide (0.67 g) in dimethylformamide (8 ml) was stirred at room temperature for 20 min. 3-Bromo-9*H*-carbazole (1.0 g, 4 mmol) was added and the mixture stirred for a further 40 min. A solution of 1-(chloromethyl)-4-fluorobenzene (0.87 g, 6 mmol) in dimethylformamide (5 ml) was added dropwise with stirring. The resulting mixture was then stirred at room temperature for 12 h and poured into water (100 ml), yielding a white precipitate. The solid product was filtered off, washed with cold water and recrystallized from EtOH, giving crystals of the title compound. Yield: 1.27 g (89.5%); m.p. 420–422 K. The title compound (40 mg) was dissolved in a mixture of chloroform (5 ml) and ethanol (5 ml) and the solution was kept at room temperature for 13 days. Evaporation of the solution gave colourless crystals suitable for X-ray analysis.

### Refinement

All H atoms were included in the idealized positions and refined in a riding model approximation with C—H distances of 0.93 (benzene) and 0.97 (methylene) Å, and with *U*_iso_(H) = 1.2x*U*_eq_(C).

### Crystal data

**Table:**

C_19_H_13_BrFN	*D*_x_ = 1.606 Mg m^−^^3^
*M**_r_* = 354.21	Melting point = 420–422 K
Orthorhombic, *P**n**a*2_1_	Mo *K*α radiation, λ = 0.71073 Å
Hall symbol: P 2c -2n	Cell parameters from 4959 reflections
*a* = 17.407 (4) Å	θ = 1.8–27.9°
*b* = 15.068 (3) Å	µ = 2.81 mm^−^^1^
*c* = 5.5865 (11) Å	*T* = 113 K
*V* = 1465.3 (5) Å^3^	Prism, colorless
*Z* = 4	0.18 × 0.12 × 0.08 mm
*F*(000) = 712	

### Data collection

**Table:**

Rigaku Saturn diffractometer	2577 independent reflections
Radiation source: rotating anode	2294 reflections with *I* > 2σ(*I*)
confocal multilayer X-ray optic	*R*_int_ = 0.050
Detector resolution: 7.31 pixels mm^-1^	θ_max_ = 25.0°, θ_min_ = 1.8°
ω and φ scans	*h* = −20→20
Absorption correction: multi-scan (*CrystalClear*; Rigaku/MSC, 2005)	*k* = −11→17
*T*_min_ = 0.632, *T*_max_ = 0.806	*l* = −6→6
9581 measured reflections	

### Refinement

**Table:**

Refinement on *F*^2^	Secondary atom site location: difference Fourier map
Least-squares matrix: full	Hydrogen site location: inferred from neighbouring sites
*R*[*F*^2^ > 2σ(*F*^2^)] = 0.032	H-atom parameters constrained
*wR*(*F*^2^) = 0.070	*w* = 1/[σ^2^(*F*_o_^2^) + (0.0325*P*)^2^] where *P* = (*F*_o_^2^ + 2*F*_c_^2^)/3
*S* = 0.99	(Δ/σ)_max_ = 0.002
2577 reflections	Δρ_max_ = 0.43 e Å^−^^3^
199 parameters	Δρ_min_ = −0.69 e Å^−^^3^
1 restraint	Absolute structure: Flack (1983), 1139 Friedel pairs
Primary atom site location: structure-invariant direct methods	Flack parameter: 0.004 (12)

### Special details

**Table:**

Geometry. All e.s.d.'s (except the e.s.d. in the dihedral angle between two l.s. planes) are estimated using the full covariance matrix. The cell e.s.d.'s are taken into account individually in the estimation of e.s.d.'s in distances, angles and torsion angles; correlations between e.s.d.'s in cell parameters are only used when they are defined by crystal symmetry. An approximate (isotropic) treatment of cell e.s.d.'s is used for estimating e.s.d.'s involving l.s. planes.
Refinement. Refinement of *F*^2^ against ALL reflections. The weighted *R*-factor *wR* and goodness of fit *S* are based on *F*^2^, conventional *R*-factors *R* are based on *F*, with *F* set to zero for negative *F*^2^. The threshold expression of *F*^2^ > σ(*F*^2^) is used only for calculating *R*-factors(gt) *etc*. and is not relevant to the choice of reflections for refinement. *R*-factors based on *F*^2^ are statistically about twice as large as those based on *F*, and *R*- factors based on ALL data will be even larger.

### Fractional atomic coordinates and isotropic or equivalent isotropic displacement parameters (Å^2^)

**Table:**

	*x*	*y*	*z*	*U*_iso_*/*U*_eq_	
Br1	0.257719 (15)	0.68839 (2)	1.21455 (15)	0.02390 (12)	
F1	0.64152 (11)	1.09557 (12)	0.7028 (5)	0.0424 (5)	
N1	0.53809 (16)	0.69284 (17)	0.5849 (5)	0.0157 (7)	
C1	0.58579 (15)	0.63539 (19)	0.7103 (7)	0.0154 (6)	
C2	0.66123 (18)	0.6094 (2)	0.6612 (6)	0.0195 (8)	
H2	0.6871	0.6304	0.5270	0.023*	
C3	0.69613 (18)	0.5514 (2)	0.8188 (6)	0.0224 (9)	
H3	0.7461	0.5328	0.7882	0.027*	
C4	0.6585 (2)	0.5198 (2)	1.0230 (7)	0.0240 (9)	
H4	0.6837	0.4815	1.1273	0.029*	
C5	0.58356 (18)	0.5459 (2)	1.0697 (6)	0.0187 (8)	
H5	0.5582	0.5247	1.2046	0.022*	
C6	0.54659 (18)	0.6037 (2)	0.9140 (6)	0.0156 (8)	
C7	0.47175 (18)	0.6440 (2)	0.9118 (6)	0.0137 (7)	
C8	0.40789 (17)	0.6400 (2)	1.0641 (5)	0.0163 (8)	
H8	0.4078	0.6034	1.1982	0.020*	
C9	0.34574 (19)	0.6916 (2)	1.0091 (6)	0.0165 (8)	
C10	0.34296 (18)	0.7470 (2)	0.8090 (6)	0.0183 (8)	
H10	0.2991	0.7804	0.7778	0.022*	
C11	0.40517 (18)	0.7520 (2)	0.6580 (6)	0.0199 (8)	
H11	0.4044	0.7896	0.5260	0.024*	
C12	0.46916 (15)	0.70008 (18)	0.7062 (8)	0.0144 (6)	
C13	0.56110 (19)	0.7523 (2)	0.3949 (6)	0.0188 (8)	
H13A	0.5193	0.7572	0.2808	0.023*	
H13B	0.6047	0.7268	0.3119	0.023*	
C14	0.58251 (18)	0.8443 (2)	0.4797 (6)	0.0136 (7)	
C15	0.62396 (17)	0.8572 (2)	0.6911 (8)	0.0210 (7)	
H15	0.6382	0.8084	0.7833	0.025*	
C16	0.64408 (18)	0.9420 (2)	0.7646 (6)	0.0253 (10)	
H16	0.6720	0.9504	0.9047	0.030*	
C17	0.6225 (2)	1.0122 (2)	0.6294 (6)	0.0258 (9)	
C18	0.5814 (2)	1.0036 (2)	0.4166 (7)	0.0280 (9)	
H18	0.5677	1.0528	0.3257	0.034*	
C19	0.56160 (18)	0.9178 (2)	0.3464 (6)	0.0211 (8)	
H19	0.5336	0.9097	0.2062	0.025*	

### Atomic displacement parameters (Å^2^)

**Table:**

	*U*^11^	*U*^22^	*U*^33^	*U*^12^	*U*^13^	*U*^23^
Br1	0.01522 (18)	0.0313 (2)	0.02516 (18)	0.00202 (13)	0.0037 (3)	−0.0041 (2)
F1	0.0644 (14)	0.0163 (10)	0.0464 (12)	−0.0111 (9)	0.0121 (17)	−0.0079 (14)
N1	0.0132 (16)	0.0153 (16)	0.0187 (16)	−0.0052 (12)	0.0024 (12)	−0.0004 (13)
C1	0.0149 (15)	0.0133 (15)	0.0180 (15)	−0.0033 (12)	−0.003 (2)	−0.004 (2)
C2	0.0160 (17)	0.0242 (19)	0.018 (2)	−0.0069 (15)	0.0010 (14)	−0.0034 (16)
C3	0.0119 (19)	0.020 (2)	0.035 (2)	0.0037 (15)	−0.0007 (16)	−0.0073 (17)
C4	0.023 (2)	0.017 (2)	0.032 (2)	0.0003 (17)	−0.0037 (17)	−0.0009 (18)
C5	0.018 (2)	0.0174 (19)	0.0203 (18)	0.0009 (16)	−0.0073 (16)	−0.0043 (16)
C6	0.0133 (18)	0.0165 (19)	0.0170 (18)	−0.0070 (15)	0.0033 (15)	−0.0080 (16)
C7	0.0151 (18)	0.0103 (18)	0.0158 (17)	0.0010 (15)	−0.0042 (14)	−0.0019 (16)
C8	0.0145 (19)	0.0160 (18)	0.0185 (18)	−0.0024 (16)	−0.0019 (14)	0.0004 (16)
C9	0.0114 (19)	0.018 (2)	0.0197 (19)	−0.0047 (15)	0.0032 (15)	−0.0067 (16)
C10	0.0132 (19)	0.0189 (19)	0.0228 (18)	0.0016 (15)	−0.0032 (14)	−0.0026 (15)
C11	0.0247 (19)	0.0155 (18)	0.020 (2)	−0.0040 (16)	−0.0033 (14)	0.0011 (15)
C12	0.0134 (14)	0.0137 (16)	0.0161 (15)	−0.0035 (12)	0.003 (2)	−0.0022 (19)
C13	0.0177 (19)	0.021 (2)	0.0177 (17)	−0.0020 (16)	0.0011 (15)	0.0000 (17)
C14	0.0124 (18)	0.0126 (18)	0.0158 (17)	−0.0036 (15)	0.0083 (14)	−0.0013 (16)
C15	0.0239 (17)	0.0190 (17)	0.0201 (18)	−0.0012 (14)	0.000 (2)	0.004 (2)
C16	0.0259 (19)	0.027 (2)	0.023 (3)	−0.0048 (16)	0.0036 (15)	−0.0061 (17)
C17	0.031 (2)	0.015 (2)	0.031 (2)	−0.0017 (17)	0.0144 (16)	−0.0045 (17)
C18	0.032 (2)	0.021 (2)	0.031 (2)	0.0086 (19)	0.0079 (19)	0.0025 (19)
C19	0.0169 (19)	0.028 (2)	0.0184 (19)	−0.0005 (17)	0.0032 (15)	0.0038 (17)

### Geometric parameters (Å, °)

**Table:**

Br1—C9	1.915 (3)	C8—H8	0.9300
F1—C17	1.363 (4)	C9—C10	1.396 (5)
N1—C12	1.382 (4)	C10—C11	1.375 (4)
N1—C1	1.389 (4)	C10—H10	0.9300
N1—C13	1.446 (4)	C11—C12	1.387 (4)
C1—C2	1.398 (4)	C11—H11	0.9300
C1—C6	1.410 (5)	C13—C14	1.512 (4)
C2—C3	1.381 (5)	C13—H13A	0.9700
C2—H2	0.9300	C13—H13B	0.9700
C3—C4	1.399 (5)	C14—C19	1.383 (4)
C3—H3	0.9300	C14—C15	1.397 (5)
C4—C5	1.387 (5)	C15—C16	1.387 (4)
C4—H4	0.9300	C15—H15	0.9300
C5—C6	1.389 (5)	C16—C17	1.353 (5)
C5—H5	0.9300	C16—H16	0.9300
C6—C7	1.437 (4)	C17—C18	1.394 (6)
C7—C8	1.401 (4)	C18—C19	1.394 (5)
C7—C12	1.427 (5)	C18—H18	0.9300
C8—C9	1.368 (5)	C19—H19	0.9300
			
C12—N1—C1	108.7 (3)	C9—C10—H10	120.1
C12—N1—C13	123.5 (3)	C10—C11—C12	118.9 (3)
C1—N1—C13	126.2 (3)	C10—C11—H11	120.6
N1—C1—C2	129.6 (3)	C12—C11—H11	120.6
N1—C1—C6	109.2 (3)	N1—C12—C11	130.3 (3)
C2—C1—C6	121.2 (3)	N1—C12—C7	108.7 (2)
C3—C2—C1	117.8 (3)	C11—C12—C7	121.0 (3)
C3—C2—H2	121.1	N1—C13—C14	114.0 (3)
C1—C2—H2	121.1	N1—C13—H13A	108.8
C2—C3—C4	121.9 (3)	C14—C13—H13A	108.8
C2—C3—H3	119.0	N1—C13—H13B	108.8
C4—C3—H3	119.0	C14—C13—H13B	108.8
C5—C4—C3	119.8 (3)	H13A—C13—H13B	107.6
C5—C4—H4	120.1	C19—C14—C15	118.7 (3)
C3—C4—H4	120.1	C19—C14—C13	120.0 (3)
C4—C5—C6	119.7 (3)	C15—C14—C13	121.3 (3)
C4—C5—H5	120.2	C16—C15—C14	120.5 (3)
C6—C5—H5	120.2	C16—C15—H15	119.7
C5—C6—C1	119.6 (3)	C14—C15—H15	119.7
C5—C6—C7	133.6 (3)	C17—C16—C15	119.0 (3)
C1—C6—C7	106.8 (3)	C17—C16—H16	120.5
C8—C7—C12	119.3 (3)	C15—C16—H16	120.5
C8—C7—C6	134.1 (3)	C16—C17—F1	119.0 (4)
C12—C7—C6	106.6 (3)	C16—C17—C18	123.1 (3)
C9—C8—C7	117.8 (3)	F1—C17—C18	117.8 (4)
C9—C8—H8	121.1	C17—C18—C19	116.9 (3)
C7—C8—H8	121.1	C17—C18—H18	121.5
C8—C9—C10	123.2 (3)	C19—C18—H18	121.5
C8—C9—Br1	118.9 (3)	C14—C19—C18	121.7 (3)
C10—C9—Br1	117.9 (3)	C14—C19—H19	119.1
C11—C10—C9	119.8 (3)	C18—C19—H19	119.1
C11—C10—H10	120.1		
			
C12—N1—C1—C2	−178.2 (3)	C9—C10—C11—C12	1.3 (5)
C13—N1—C1—C2	−12.3 (5)	C1—N1—C12—C11	176.9 (3)
C12—N1—C1—C6	1.0 (3)	C13—N1—C12—C11	10.5 (5)
C13—N1—C1—C6	166.9 (3)	C1—N1—C12—C7	−1.6 (3)
N1—C1—C2—C3	179.3 (3)	C13—N1—C12—C7	−167.9 (3)
C6—C1—C2—C3	0.1 (5)	C10—C11—C12—N1	−179.9 (3)
C1—C2—C3—C4	−0.7 (5)	C10—C11—C12—C7	−1.7 (5)
C2—C3—C4—C5	0.9 (5)	C8—C7—C12—N1	−180.0 (3)
C3—C4—C5—C6	−0.5 (5)	C6—C7—C12—N1	1.5 (4)
C4—C5—C6—C1	−0.1 (5)	C8—C7—C12—C11	1.4 (5)
C4—C5—C6—C7	−178.7 (3)	C6—C7—C12—C11	−177.1 (3)
N1—C1—C6—C5	−179.0 (3)	C12—N1—C13—C14	72.0 (4)
C2—C1—C6—C5	0.3 (5)	C1—N1—C13—C14	−92.0 (4)
N1—C1—C6—C7	−0.1 (4)	N1—C13—C14—C19	−141.1 (3)
C2—C1—C6—C7	179.2 (3)	N1—C13—C14—C15	39.5 (4)
C5—C6—C7—C8	−0.4 (7)	C19—C14—C15—C16	−0.4 (5)
C1—C6—C7—C8	−179.1 (4)	C13—C14—C15—C16	179.1 (3)
C5—C6—C7—C12	177.9 (4)	C14—C15—C16—C17	0.5 (5)
C1—C6—C7—C12	−0.8 (4)	C15—C16—C17—F1	179.3 (3)
C12—C7—C8—C9	−0.8 (5)	C15—C16—C17—C18	−0.7 (5)
C6—C7—C8—C9	177.3 (3)	C16—C17—C18—C19	0.8 (5)
C7—C8—C9—C10	0.5 (5)	F1—C17—C18—C19	−179.2 (3)
C7—C8—C9—Br1	−179.6 (2)	C15—C14—C19—C18	0.5 (5)
C8—C9—C10—C11	−0.8 (5)	C13—C14—C19—C18	−178.9 (3)
Br1—C9—C10—C11	179.4 (2)	C17—C18—C19—C14	−0.7 (5)
